# A rare cause of heart failure

**DOI:** 10.1002/ccr3.1543

**Published:** 2018-04-22

**Authors:** Dominika Zoltowska, Jagadeesh Kumar Kalavakunta

**Affiliations:** ^1^ Department of Internal Medicine Western Michigan University Homer Stryker School of Medicine 300 Portage Street Kalamazoo 49007 Michigan; ^2^ Department of Cardiology Michigan State University/Borgess Medical Center 1521 Gull Rd Kalamazoo 49048 Michigan

**Keywords:** Cardiovascular disease, echocardiography, heart failure, noncompaction cardiomyopathy

## Abstract

Noncompaction cardiomyopathy (NCCM) is a rare but important cause of heart failure. The reported prevalence of left ventricle NCCM based on echocardiographic criteria varies between 0.014% and 1.3%, while the biventricular involvement is extremely rare with only few cases reported.

## Case

A 47‐year‐old male patient presented with new onset shortness of breath and bilateral lower extremity swelling. His past medical history comprised hypertension and end‐stage renal disease on regular hemodialysis. Vital signs were within normal limit and physical examination was significant for bilateral pitting edema below the knee, 2+. Laboratory tests showed elevated B‐type natriuretic peptide at 4000 pq/mL and negative troponin. No ischemic changes were registered on electrocardiogram. Transthoracic echocardiogram (TTE) findings were consistent with biventricular noncompaction cardiomyopathy (Videos S1–S3). Differential diagnosis comprised hypertrophic obstructive cardiomyopathy (HOCM), hypertensive heart disease, and infiltrative cardiomyopathies such as amyloidosis and sarcoidosis; 6 L of fluid was removed via ultrafiltration, and the patient was discharged in stable condition on beta blocker and ACE inhibitor. Deemed the high risk of arrhythmia, patient was referred for evaluation for implantable cardioverter‐defibrillator (ICD) placement for primary prevention of sudden cardiac death.

## Question: What are Echocardiographic Criteria for Noncompaction Cardiomyopathy?

Jenni criteria are commonly utilized. Accordingly, TTE should show bilayered myocardium with multiple, prominent trabeculations in end‐systole and a ratio of noncompacted to compacted myocardium >2:1. Color Doppler needs to reveal flow within the deep intertrabecular recesses and prominent trabecular meshwork 1. Contrast‐enhanced echocardiography is helpful to discriminate the compacted and the noncompacted myocardium. For further differentiation, the utilization of magnetic resonance imaging and computed tomography may be warranted. This could be the additional step in our case.

## Authorship

DZ and JK: were the physicians seeing the patient in the clinic. DZ and JK: were responsible for performing, diagnosing, and discussing the imaging studies of the patient. DZ: prepared the manuscript draft, which was critically revised and approved by JK.

## Conflict of Interest

None declared.

## Supporting information


**Video S1**. Transthoracic echocardiogram. Left ventricle.

**Video S2**. Transthoracic echocardiogram. Four‐chamber view.

**Video S3**. Transthoracic echocardiogram. Subxiphoid view with Doppler study.
